# Supplementary material for: A Protocol to Self-Familiarize Health Care Professionals with the Detection Limits of a Physical Activity Tracker for Low-Impact Steps in Patients Recovering from Knee Surgery—A Proposal and a First Evaluation
Source: Sensors (Basel). 2025 Nov 1;25(21):6666. doi: 10.3390/s25216666 (PMC12608956; doi:10.3390/s25216666)
Supplement: Supplementary file 1 [file sensors-25-06666-s001.zip › SupplementalFile S1.pdf]

Supplementary File S1: The PATs included in this study

The PATs were selected according to the following criteria:

- They should be commercially available or represent prototypes in the final stages of commercialisation.
- They should have a battery time that allows monitoring of physical activities over several days in a home setting without the need for recharging.
- They should represent a variety of measurements locations (e.g. wrist, waist, knee, foot) and/or measurements technologies, as this probably leads to differences in sensitivity for low-impact steps.
- They should allow an immediate inspection of the step counts after performing 10 to 20 steps.

The following four PATs were included in this study:

STAPPONE Rehab sole sensor

This is a commercially available sensor developed to monitor the weight bearing of patients, in particular with respect to recommendations on restricting weight bearing.

STAPPONE Rehab offers a continuous monitoring without recalibration over 6 hours. However, according to the manufacture the sensor can be adapted to support longer measurement periods.

The sensor provides information on step counts over short time intervals directly on the accompanying app.

The sensor is based on a 4×6-axis IMU, 4×3-axis acceleration, 2×3-axis magnetometer and 2×3-axis gyroscope with a sampling rate of 50-100Hz.

Further information can be found at https://www.stappone.com/en/technology/.

Orthronic Smart Knee sensor

This sensor consists of two parts which have to be attached above and below the knee. The sensor measures the knee angle continuously in time. The sensor allows measurements up to 10 days.

The sensor is developed by the company Stat Consult. It is not yet commercially available but is intended to be offered commercially to HCPs. It is already in use in one clinical research study (Mba et al. *Sensors* **2025**, *25*, 118. https://doi.org/10.3390/s25010118)

The inertial measurement unit incorporates a 3-axis accelerometer, gyroscope, and magnetometer. The sampling rate is 25 Hz.

An app allows inspection of knee angle measurements as a time series in quasi-continuous time, resulting in a curve similar to a sinus wave. The number of steps can be determined manually by counting the number of peaks.

OMRON Walking Style IV

This is a traditional step counter offered by a company specialized in medical devices. It can be worn in a trouser pocket, attached to a cord around the neck, or in a bag.

The steps are counted continuously and shown on a display. According to the instructions of the manufacturer, steps are only counted after 4 seconds of walking.

The battery allows measurements over 1 year.

Further information can be found at https://www.omron-healthcare.com/products/walking-style-iv-blue

Fitbit inspire 2

This is a popular fitness tracker with a battery time of 10 days. It is worn at the wrist.

The tracker has a small screen showing the current time and one additional parameter. The parameter can be selected to be the actual step count.

Tracking of motion patterns is based on a 3-axis accelerometer.

At the timepoint of submission of the paper, the tracker was no longer commercially available. Information could be still found at https://www.fitbit.com/content/assets/help/manuals/manual_inspire_2_en_US.pdf.
