# Supplementary material for: A Protocol to Self-Familiarize Health Care Professionals with the Detection Limits of a Physical Activity Tracker for Low-Impact Steps in Patients Recovering from Knee Surgery—A Proposal and a First Evaluation
Source: Sensors (Basel). 2025 Nov 1;25(21):6666. doi: 10.3390/s25216666 (PMC12608956; doi:10.3390/s25216666)
Supplement: Supplementary file 1 [file sensors-25-06666-s001.zip › SupplementalFile S2.pdf]

Supplementary File S2: Definition of agreement rate

In the context of this study, step count ratios mainly distinguish three possible outcome scenarios for a specific experimental condition and a specific PAT:

1. The PAT is able to identify steps under this condition. In this case, we expect a ratio close to 1.0. We cannot expect that the ratio is exactly 1.0, as there is some uncertainty in the experimental set up. Initial steps may differ from a steady state walking (reference) and/or may systematically be neglected by a PAT, and "half" steps at the start and end of each walking episode may or may not be counted.
2. The PAT is unable to identify steps under this condition. In this case, we expect a ratio close to 0.0. We cannot expect that the ratio is exactly 0.0, as the PAT may still generate a few counts reflecting other types of movements.
3. The experimental condition is close to the limit, at which the PAT can detect step counts. In this case we have to expect that the step count ratio is rather unstable and may cover a rather wide range of values.

Consequently, whereas under scenario 1 and 2 we can expect to reproduce step count ratios within rather small ranges as indicated above, this is not the case for scenario 3. Indeed, the whole set up of the protocol and the study aims mainly at distinguishing between scenario 1 and scenario 2. To focus on the reproducibility in distinguishing between these two scenarios, weighted agreement rates were considered. In determining the degree of agreement between two single step count ratios, the following weights were used:

0	If one step count ratio is above 0.8 and the other is below 0.2
0.5	If one step count ratio is between 0.5 and 0.8 and the other is below 0.2 or if one step count ratio is between 0.2 and 0.5 and the other is above 0.8
1	If both step count ratios are between 0.2 and 0.8 or if one step count ratio is above 0.8 and the other above 0.5 or if one step count is below 0.2 and the other below 0.5.

The agreement rate of a set of pairs of step count ratios was then defined as the average weight over all pairs. The interpretation of agreement rates depends on the distribution of the step-count ratios involved: If the variation of step count ratios is limited, it is easy to obtain a high agreement, and with increasing variation this becomes more challenging. To facilitate the interpretation of agreement rates we followed the idea of Cohen's κ [18] and compared the observed agreement rate with the expected agreement rate under chance conditions. The latter was computed by permuting the first element of each pair of step count ratios across all pairs, computing the agreement rate for all permutations, and taking the average. The observed agreement rate a and the

expected agreement rate e were then combined to a kappa value using Cohen's formula, i.e. $\kappa = (a - e)/(1 - e)$.
