# Supplementary material for: A Protocol to Self-Familiarize Health Care Professionals with the Detection Limits of a Physical Activity Tracker for Low-Impact Steps in Patients Recovering from Knee Surgery—A Proposal and a First Evaluation
Source: Sensors (Basel). 2025 Nov 1;25(21):6666. doi: 10.3390/s25216666 (PMC12608956; doi:10.3390/s25216666)
Supplement: Supplementary file 1 [file sensors-25-06666-s001.zip › SupplementalTable S1.pdf]

Trouser sensor			
	estimate	95%-CI	p-value
S linear	-6.3	[-7.4,-5.3]	<0.001
T vs S	-0.8	[-3.8,2.2]	0.586
T-75 vs T-40	-9.4	[-13.8,-4.9]	<0.001
Z vs S	-3.7	[-7.0,-0.4]	0.028
Z-75 vs Z-40	-6.3	[-11.0,-1.5]	0.010
C vs S	0.2	[-2.9,3.4]	0.884
C-75 vs C-40	-12.4	[-15.4,-9.4]	<0.001
W vs S	-7.7	[-10.7,-4.6]	<0.001
W-75 vs W-40	-8.0	[-12.0,-4.1]	<0.001

Wrist sensor			
	estimate	95%-CI	p-value
S linear	-2.8	[-4.3,-1.3]	<0.001
T vs S	-0.8	[-2.3,0.7]	0.286
T-75 vs T-40	-1.8	[-4.0,0.5]	0.123
Z vs S	-0.2	[-2.0,1.6]	0.797
Z-75 vs Z-40	-0.6	[-2.8,1.6]	0.605
C vs S	-0.8	[-2.5,0.9]	0.382
C-75 vs C-40	-4.4	[-8.3,-0.4]	0.032
W vs S	0.3	[-2.8,3.4]	0.834
W-75 vs W-40	-5.3	[-10.2,-0.4]	0.035

Supplemental Table S1: Estimates of selected contrasts together with 95% confidence intervals and p-values.
