# Supplementary figures and images for: A Protocol to Self-Familiarize Health Care Professionals with the Detection Limits of a Physical Activity Tracker for Low-Impact Steps in Patients Recovering from Knee Surgery—A Proposal and a First Evaluation
Source: Sensors (Basel). 2025 Nov 1;25(21):6666. doi: 10.3390/s25216666 (PMC12608956; doi:10.3390/s25216666)

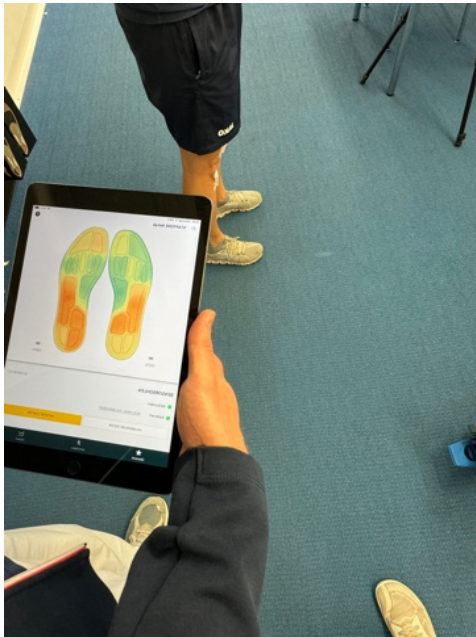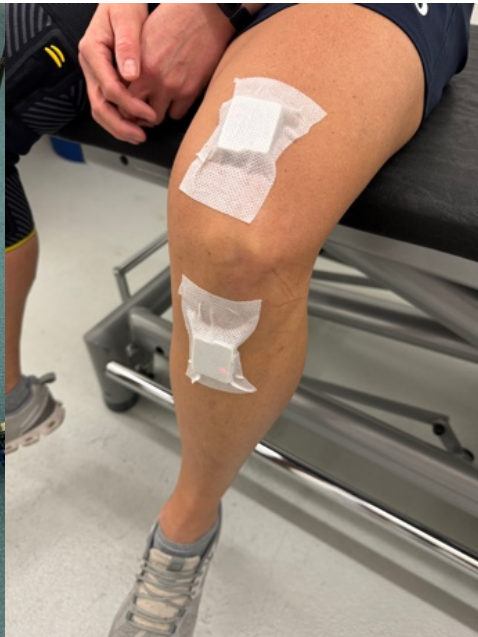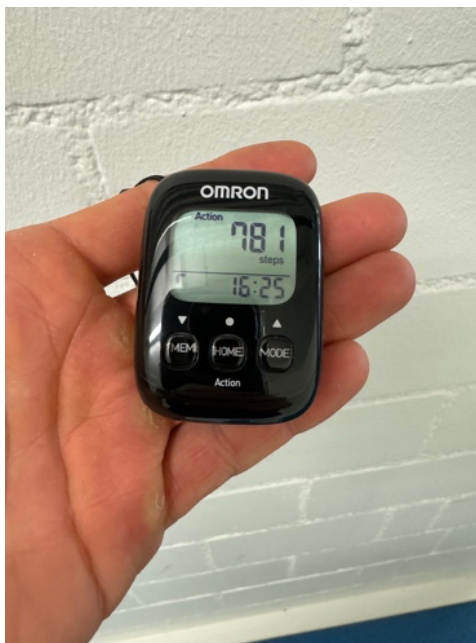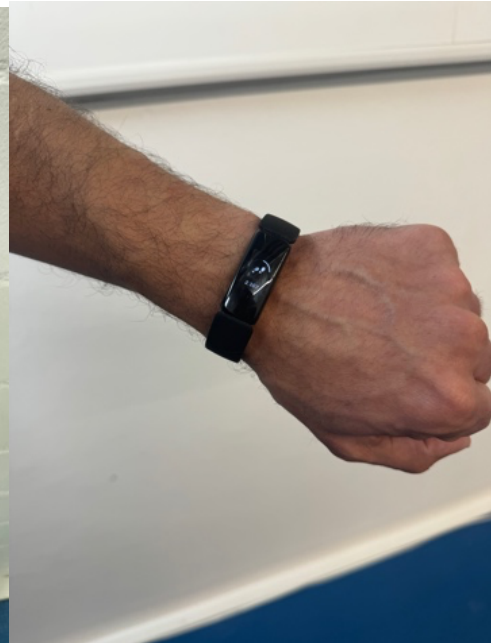

Supplement: Supplementary file 1 [file sensors-25-06666-s001.zip › SupplementalFigure S1.pdf]

step count ratio

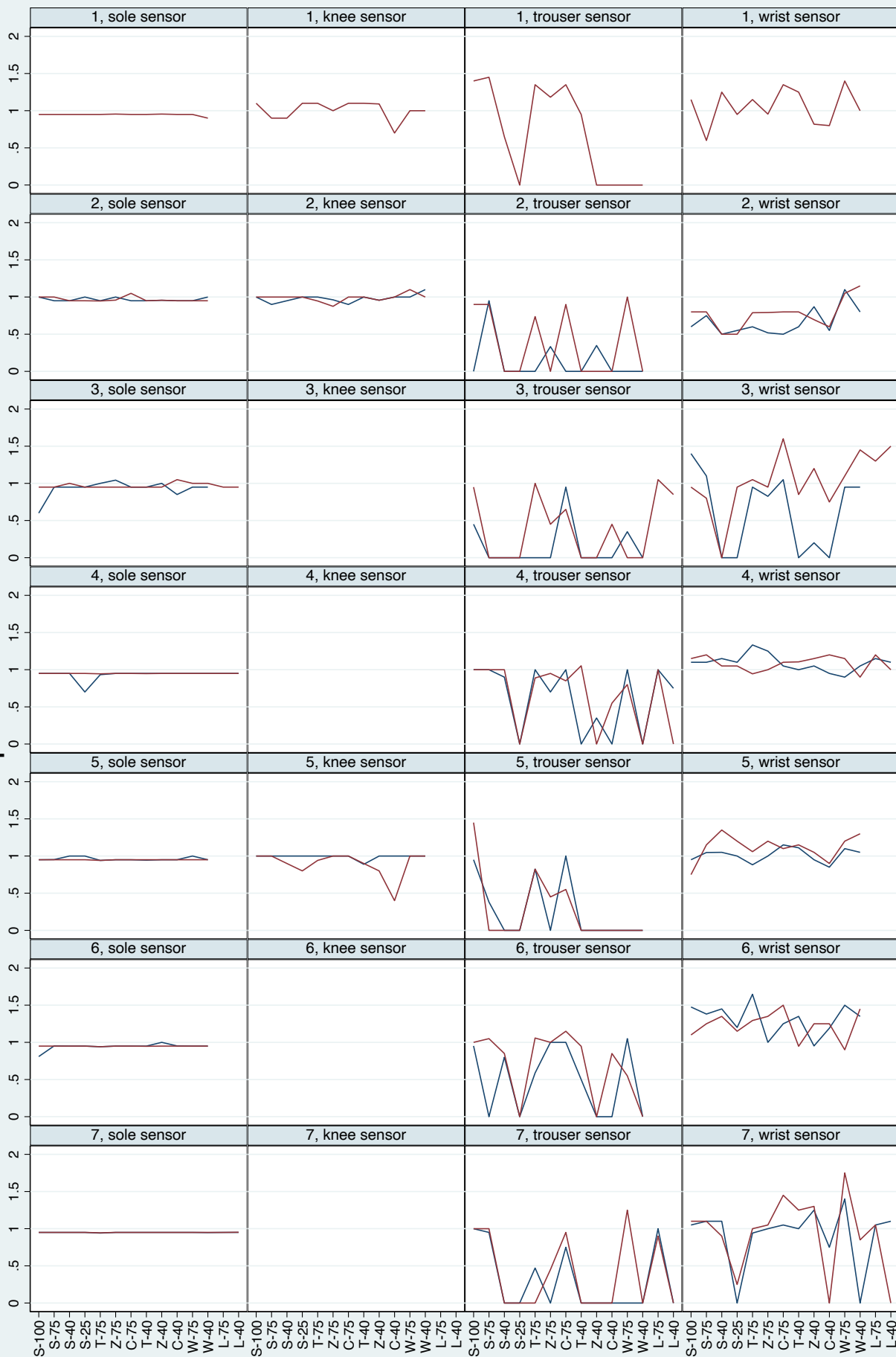

Supplement: Supplementary file 1 [file sensors-25-06666-s001.zip › SupplementalFigure S2.pdf]

step count ratio

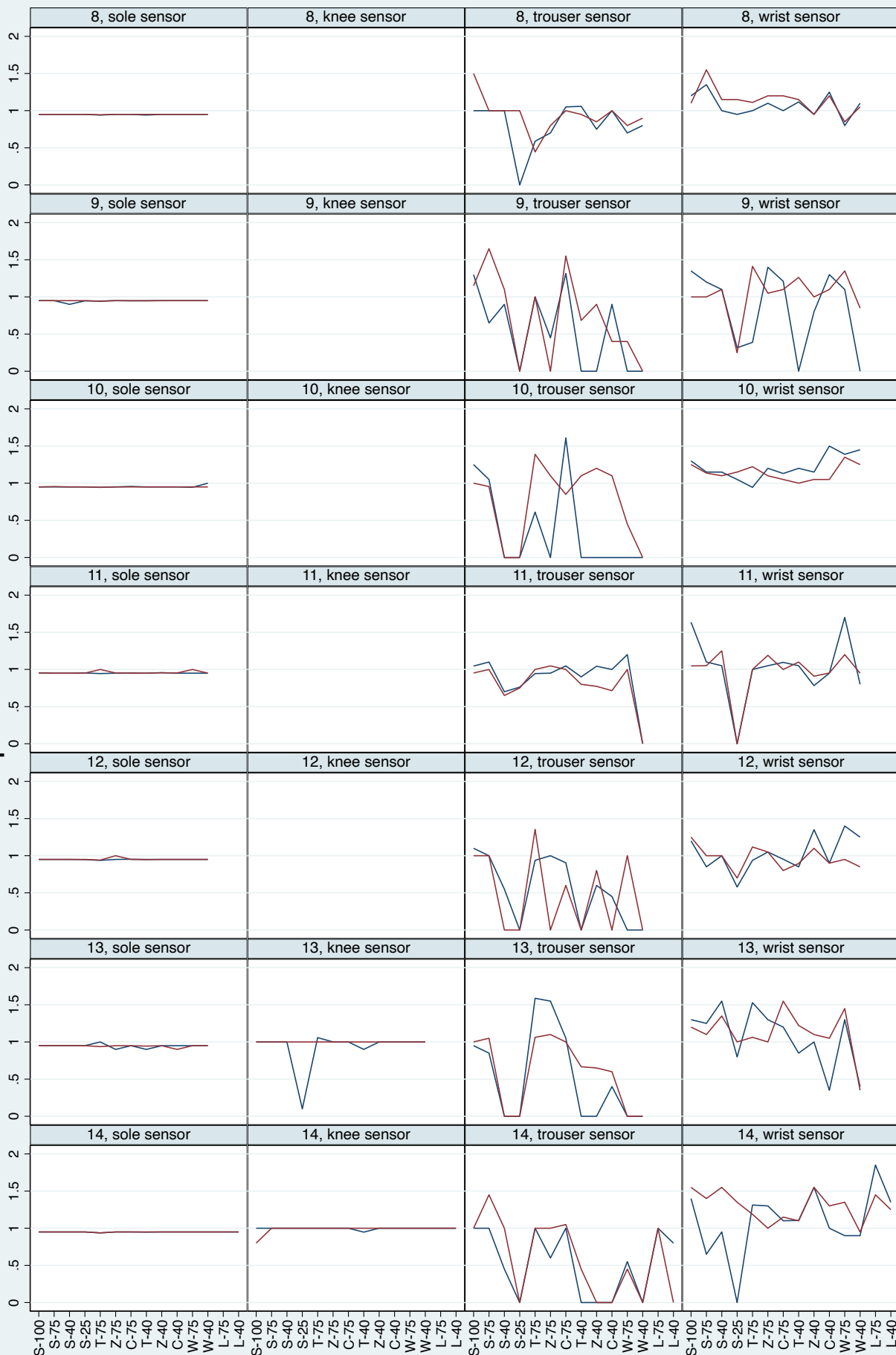

Supplement: Supplementary file 1 [file sensors-25-06666-s001.zip › SupplementalFigure S3.pdf]
